# Education is an important factor in end-of-life care: results from a survey of Brazilian physicians’ attitudes and knowledge in end-of-life medicine

**DOI:** 10.1186/s12909-020-02253-8

**Published:** 2020-10-02

**Authors:** Thais Ioshimoto, Danielle Ioshimoto Shitara, Gilmar Fernades do Prado, Raymon Pizzoni, Rafael Hennemann Sassi, Aécio Flávio Teixeira de Gois

**Affiliations:** 1 Departamento de Medicina. Rua Pedro de Toledo, Universidade Federal de São Paulo (UNIFESP), 719, São Paulo, SP Brazil; 2 Hospital Israelita Albert Einstein (HIAE), São Paulo, SP Brazil

**Keywords:** Education, End-of-life care, Knowledge, Palliative care knowledge test (PCKT), Palliative medicine, Medical residency, Developing countries

## Abstract

**Background:**

According to the Latin America Association for palliative care, Brazil offers only 0.48 palliative care services per 1 million inhabitants. In 2012, no accredited physicians were working in palliative care, while only 1.1% of medical schools included palliative care education in their undergraduate curricula. As a reflection of the current scenario, little research about end-of-life care has been published so that studies addressing this subject in the Brazilian setting are crucial.

**Methods:**

A cross-sectional study study conducted with students applying for the medical residency of the Federal University of São Paulo were invited to voluntarily participate in an anonymous and self-administered questionnaire survey. The latter included demographic information, attitudes, prior training in end-of-life care, prior end-of-life care experience, the 20-item Palliative Care Knowledge Test (PCKT) and a consent term.

**Results:**

Of the 3086 subjects applying for residency, 2349 (76%) answered the survey, 2225 were eligible for analysis while 124 were excluded due to incomplete data. Although the majority (99,2%) thought it was important to have palliative care education in the medical curriculum, less than half of them (46,2%) reported having received no education on palliative care. The overall performance in the PCKT was poor, with a mean score of 10,79 (± 3). While philosophical questions were correctly answered (81,8% of correct answers), most participants lacked knowledge in symptom control (50,7% for pain, 57,3% for dyspnea, 52,2% for psychiatric problems and 43,4% for gastrointestinal problems). Doctors that had already concluded a prior residency program and the ones that had prior experience with terminal patients performed better in the PCKT (*p* < 0,001). The high-performance group (more than 50% of correct answers) had received more training in end-of-life care, showed more interest in learning more about the subject, had a better sense of preparedness, as well as a higher percentage of experience in caring for terminal patients (*p* < 0,001).

**Conclusions:**

Our study showed that Brazilian physicians lack not only the knowledge, but also training in end-of-life medicine. Important factors to better knowledge in end-of-life care were prior training, previous contact with dying patients and prior medical residency. Corroborating the literature, for this group, training showed to be a key factor in overall in this area of knowledge. Therefore, Brazilian medical schools and residency programs should focus on improving palliative training, especially those involving contact with dying patients.

## Key questions

**What is already known about the topic?**

Very little research in end-of-life care has been published in developing countries. In Brazil, there is no data about end-of-life medicine knowledge and training among physicians. In the U.S. medical schools, there are currently no universal, standardized medical curricula and clinical training protocols dedicated to palliative medicine. Studies assessing end-of-life knowledge among students and physicians in developed countries are not representative of the overall reality due to small samples and local studies.

**What does this paper add?**

This study demonstrates that Brazilian physicians lack training and knowledge in end-of-life medicine. It also shows that training is considered to be a key factor in overall end-of-life care knowledge.

**Implications for practice, theory or policy?**

The study is an important wake-up call for the importance of palliative care training in Brazilian medical schools and residency programs. They should focus on improving end-of-life training, especially those involving contact with terminal patients. We hope that this study will induce a change in the Brazilian medical curricula.

## Background

The current status of palliative care in developing countries is alarming [1]. According to the Latin America Association for Palliative Care, Brazil offers only 0.48 palliative care services per 1 million inhabitants [2]. In 2012, no accredited physicians were working in palliative care [2]. The education scenario is also of some concern. While only 1.1% of medical schools include palliative care education in undergraduate curricula, the demand for palliative care in developing countries is steadily growing [3, 4].

The demographic transition in developing countries led to a shift in causes of death, with communicable diseases no longer being the main cause in many Latin America countries. As life expectancy increases, the prevalence of multiple chronic conditions among elderly people rises [5]. Those with advanced chronic disease will face the same palliative care problems as cancer patients, with clear indication of palliative symptoms relief [6]. A study conducted in Spain in 2010, found out that the prevalence of the advanced chronic disease in the elderly can reach 10.9% [7]. When the risk of developing non communicable diseases is evaluated, the socio-economic status of the country has a main role [8], putting the Latin American countries in the spotlight of the problem. By the year 2050, life expectancy in Brazil is estimated to be eighty years old [9]. Therefore, the number of elderly in need of palliative services will grow exponentially [10, 11].

Very little research in palliative care has been published in developing countries and applying developed countries’ perspectives on end-of-life care in developing nations is unrealistic and tends to failure [12]. Therefore, research regarding palliative care and end-of-life care in developing countries, such as Brazil, is mandatory to assess needs and priorities, establish health interventions and increase overall medical knowledge.

The purpose of our survey is to assess medical knowledge in end-of-life care to identify key factors that could be useful in improving palliative care in Brazilian medical schools and residency.

## Methods

To asses the end-of-life palliative care knowledge among 3086 students that underwent the integrated exam for all medical residency vacancies of the Federal University of São Paulo, a cross-sectional study was proposed. The applicants were invited to voluntarily participate in the survey on the exam day and gave their written consent. They were asked to answer a self-administered questionnaire assessing their knowledge, experience, sense of preparedness and training with terminally ill patients. A cover letter explained the purpose of the study and elicited that it was anonymous and confidential. Consent to participate in the study was indicated by the fulfillment and return of the questionnaire. The survey was approved by the research ethics committee of the Federal University of São Paulo and the Residency Program Director, who was responsible for the exam (CAAE 36437614.2.0000.5505).

### Questionnaire

The questionnaire consisted of three parts. The first part included demographic information: age, gender, religion, year of graduation, the medical school of graduation, prior residency program and applying medical specialty.

The second part assessed attitudes and experience in end-of-life palliative care: previous training, interest in learning more about the subject, number of terminal patients cared for and sense of preparedness in caring for this kind of patients.

The third part was a 20 item, true or false knowledge test, adapted from the Palliative Care Knowledge Test (PCKT) (Nakasawa et al.) [13, 14] to reflect the Brazilian context; this test was chosen because allows to asses not only the philosophical and technical skills but also can be used to improve the educational programs [13].

### Statistical analyses

Participants who responded to 90% or more of the 20 item questionnaire were included as valid subjects of the analyses [13]. Unsure responses were regarded as incorrect.

For the continuous variables, the mean and standard deviation were calculated. Frequency and proportion were used for categorical variables.

A comparison of categorical variables among groups was performed using the chi-square test. If necessary, the paired difference test was used. Comparison of continuous variables among groups were performed with analysis of variance (ANOVA). A *p*-value of less than 0,05 was considered significant.

### Group analysis

To analyze which factors influenced knowledge, the sample was divided into four groups according to the number of correct answers: group 1 (excellent) 76–100% of correct answers, group 2 (good) 51–75%, group 3 (poor) 26–50% and group 4 (bad) 0–25%.

## Results

Of the 3086 subjects applying for residency, 2349 (76%) answered the survey, 2225 were eligible for analysis while 124 were excluded due to incomplete data.

### Demographic data

Overall, the mean age of the participants was 26.7 years old (±2.8); 56.9% were female; 63.5% were catholic; 52.4% had attended public universities. Less than half of the subjects (38,6%) had already concluded a prior residency program, being mostly in internal medicine or clinical specialties (52.6%) Table 3.

### Education and attitudes

Over 50 % of the responders reported having had prior training in palliative care, most of them during graduation (66,9%). The ones that attended private schools had a higher percentage of training (57% versus 51% of the students in public). Almost the totality of the subjects (99.1%) agreed that palliative care is the best treatment for terminal patients, that it is important to have palliative care education in the medical curriculum (99.2%) and had an interest in learning more about the subject (94.6%). Although 73.7% had prior contact with terminal patients, the majority felt unprepared to provide good care for this sort of patient (77.2%) Table 1.

**Table 1:** Response rates regarding training and attitudes in palliative care

	Total
**Prior training in Palliative Care?**	
Yes	1194 (53.8%)
**When?**	
During endergraduation	779 (66.9%)
During Medical Residency	314 (27%)
Both	71 (6.1%)
**Do you find it is an important subject?**	
Yes	2198 (99.2%)
**Do you have interest in learning more?**	
Yes	2097 (94.6%)
**Do you fell prepared to care for dying patients?**	
Yes	504 (22.8%)
**Did you have prior experience with dying patients?**	
Yes	1639 (73.7%)

### Knowledge

The mean score of the PCKT was 10.79 (± 3), a hit rate of 54%. The percentage of correct answers was higher for philosophy (81.8%), followed by pain (50.7%), dyspnea (57.3%), psychiatric problems (52.2%) and gastrointestinal problems was (43.4%) Table 2.

**Table 2:** The Palliative Care Knowledge Test (PCKT)

	Incorrect	Correct
Philosophy		
1. Palliative care should be provided for patients who have no curative treatments available.	549 (24.7%)	1676 (75.3%)
2. Palliative care should not be provided along with other anti-cancer treatments.	262 (11.8%)	1963 (88.2%)
TOTAL for philosophy	18.2%	81.8%
Pain		
3. One of the goals of pain management is to get a good night’s sleep.	265 (11.9%)	1960 (88.1%)
4. When cancer pain is mild, pentazocine should be used more often than an opioid.	1217 (54.7%)	1008 (45.3%)
5. When opioids are taken on a regular basis, non-steroidal anti-inflammatory drugs should not be used.	766 (34.4%)	1459 (65.6%)
6. The effect of opioids should decrease when pentazocine ou buprenorphine hydrocloride is used together after opioids are used.	1733 (77.9%)	492 (22.1%)
7. Long-term use of opioids can often induce addiction.	1657 (74.5%)	568 (25.5%)
8. Use of opioids does not influence survival time.	942 (42.3%)	1283 (57.7%)
TOTAL for pain	49.3%	50.7%
Dyspnea		
9. Morphine should be used to relieve dyspnea in cancer patients	1177 (52.9%)	1048 (47.1%)
10. When opioids are used on a regular basis, respiratory depression Will be common.	1120 (50.3%)	1105 (49.7%)
11. Oxygen saturation levels are correlated with dyspnea	744 (33.4%)	1481 (66.6%)
12. Anticholinergic drugs or scopolamine hydrobromide are effective for alleviating bronchial secretions of dying patients.	766 (34.4%)	1459 (65.6%)
TOTAL for dyspnea	42.7%	57.3%
Psychiatric problems		
13. During the last days of life, drowsiness associated with electrolyte imbalance should decrease patient discomfort.	1352 (60.8%)	873 (39.2%)
14. Benzodiazepines should be effective for controlling delirium.	1039 (46.7%)	1186 (53.3%)
15. Some dying patients will require continuous sedation to alleviate suffering.	289 (13%)	1936 (87%)
16. Morphine is often a cause of delirium in terminally ill cancer patients	1573 (70.7%)	652 (29.3%)
TOTAL for psychiatric problems	47.8%	52.2%
Gastrointestinal Problems		
17. At terminal stages of cancer, higher calorie intake is needed compared to early stages.	1280 (57.5%)	945 (42.5%)
18. There is no route except central venous for patients unable to maintain a peripheral intravenous route	1442 (64.8%)	783 (35.2%)
19. Steroids should improve appetite among patients with advanced cancer	1282 (57.6%)	943 (42.4%)
20. Intravenous infusion will not be effective for alleviating dry mouth in dying patients.	1035 (46.5%)	1190 (53.5%)
TOTAL for Gastrointestinal Problems	56.6%	43.4%

### Group analyses

**Fig. 1:**
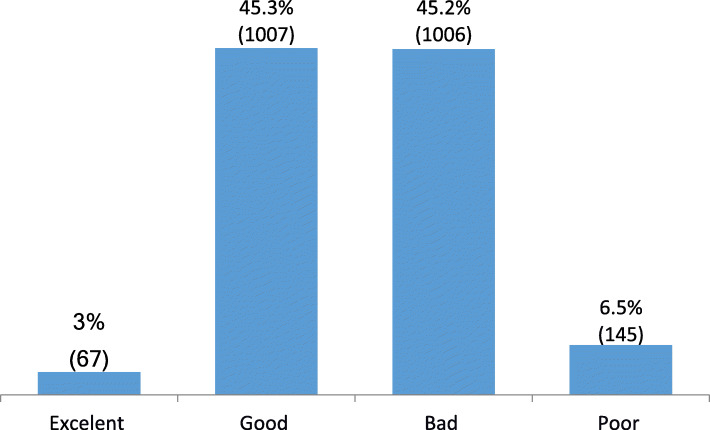
Distribution of Groups according to Percentage of correct answers: Group excellent 76–100%, Good 51–75%, Poor 26–50%, Bad 0–25%

Only 3% scored more than 75% of the test (Group 1- excellent), while more than half of the subjects scored less than 50% of the test. Figure 1 The distribution of groups followed a Gaussian pattern with the “excellent” group somewhat greater than the “poor” group. The responders in the four groups did not differ significantly in terms of age, religion, interest and given the importance of the subject. Nevertheless, group 3 (poor) had more females than other groups (*p* < 0,0001) and groups 3 and 4 (poor and bad) had more students from private schools (*p* < 0,001). Groups 1 and 2 (excellent and good) had more prior training in palliative care, showed more interest in learning more about the subject, had a higher percentage of experience, as well felt more prepared to care for terminal patients Table 3.

**Table 3:** Group Analysis according to the percentage of correct answers^a^

	Groups^a^				Total	***p***-value
	Group 1 (Bad)	Group 2 (Poor)	Group 3 (Good)	Group 4 (Excelent)		
**Age**						
Mean (SD)	26.7 (4.6)	26.5 (2,7)	26.7 (2.5)	26.8 (2.7)	26.6 (2.7)	0.2
**Graduation Medical School**						
Private	45 (67.2%)	529 (52.7%)	440 (43.9%)	41 (28.5%)	1055 (47.6%)	**< 0.0001**
Public	22 (32.8%)	474 (47.3%)	562 (56.1%)	103 (71.5%)	1161 (52.4%)	
**Prior Medical Residency**						
Yes	8 (11.9%)	257 (25.5%)	478 (47.6%)	115 (79.3%)	858 (38.6%)	**< 0.0001**
No	59 (88.1%)	749 (74.5%)	527 (52.4%)	30 (20.7%)	1365 (61.4%)	
**Medical Specialty**						
Internal Medicine	2 (28.6%)	69 (27.4%)	300 (63.3%)	104 (92%)	475 (56.1%)	**< 0.0001**
Surgery	3 (42.9%)	102 (40.5%)	101 (21.3%)	7 (6.2%)	213 (25.2%)	
Pediatrics	2 (28.6%)	81 (32.1%)	71 (15%)	2 (1.8%)	156 (18.4%)	
Others	0 (0%)	0 (0%)	2 (0.4%)	0 (0%)	2 (0.2%)	
**Prior training in Palliative Care?**						
Yes	24 (35.8%)	479 (47.7%)	577 (57.4%)	114 (79.2%)	1194 (53.8%)	**< 0.0001**
No	43 (64.2%)	525 (52.3%)	428 (42.6%)	30 (20.8%)	1026 (46.2%)	
**Do you have interest in learning more?**						
Yes	59 (89.4%)	939 (93.5%)	959 (95.6%)	140 (97.2%)	2097 (94.6%)	**0.0213**
No	7 (10.6%)	65 (6.5%)	44 (4.4%)	4 (2.8%)	120 (5.4%)	
**Do you fell prepared to care for dying patients?**						
Yes	6 (9.1%)	173 (17.2%)	240 (24%)	85 (59%)	504 (22.8%)	**< 0.0001**
No	60 (90.9%)	830 (82.8%)	759 (76%)	59 (41%)	1708 (77.2%)	
**Did you have prior experience with dying patients?**						
Yes	39 (58.2%)	708 (70.3%)	761 (75.6%)	131 (90.3%)	1639 (73.7%)	**< 0.0001**
No	28 (41.8%)	299 (29.7%)	245 (24.4%)	14 (9.7%)	586 (26.3%)	

^a^Percentage of correct answers: Group1 (excellent) 76–100%, Group 2 (Good) 51–75%, Group 3 (Poor) 26–50%, Group 4 (Bad) 0–25%

Subjects that had already concluded a prior residency program and the ones that had prior experience with terminal patients performed better at the PCKT (*p* < 0,001). Previous medical residency in internal medicine and clinical specialties had higher scores in the PCKT (*p* < 0,001) compared to surgical and pediatric residencies Table 4.

**Table 4:** Palliative Knowledge (% of correct answers) according to prior medical residency, medical specialty and prior experience with dying patients

	N	Mean (SD)	Median	Min-Maximum	***p***-value
**Prior Medical Residency?**					
No	1365	50.1 (13.3)	50	10–90	**< 0.0001**
Yes	858	60 (15.4)	60	15–100	
**Medical Specialty**					
Surgery	629	50.5 (12.9)	50	10–85	**< 0.0001**
Internal Medicine	898	59.1 (15.9)	60	10–100	
Others	696	50.4 (13.5)	50	15–90	
**Prior experience with dying patients?**					
No	586	50.5 (14.1)	50	15–90	**< 0.0001**
Yes	1639	55.1 (15.1)	55	10–100	

## Discussion

With the increase in life expectancy, people are living more and developing different kinds of diseases. The study of end-of-life care is essential in this scenario. To provide a better patient care, there is a necessity to identify how much the doctors know about this subject. This study, as far as we know, is the first one conducted in Brazil to approach newly graduated doctors’ knowledge.

The PCKT test not only measures the medical knowledge but also allow us to evaluate different areas as philosophy, enabling a multi-professional view of the patient. This kind of study facilitates a change from the old model of care centered in the doctor to a multi-professional one, centred in the patient. Thus, the results showed a poorer performance in the test contrasting with a higher interest in learning more about end-of-life and palliative care points to a necessity of a compulsory discipline in the medical curriculum.

When the results are analysed, the type of medical residency, prior training in this area and previous experience with terminal patients were important factors in overall palliative knowledge. The mean test score of our study population was inferior to what has been reported in the literature. Schroder et al. reported a mean score of 64,6% in a multicenter study that assessed internal medicine residents [15]. In Japan, the mean PCKT score was 72% of correct answers among physicians. Japanese doctors not only performed better in philosophical questions but also had good knowledge in treating pain and gastrointestinal symptoms [16]. In Turkey, physicians performed better in philosophical questions but lacked knowledge in symptoms control [17], in accordance with the findings of our research. The poor performance of Brazilian doctors seems to be a reflex of the Latin America palliative scenario. Since this study points the importance of end-of-life care education and the lack of knowledge of undergratuate and of graduate doctors, it can not only benefits Brazilian doctors and medical studentl but also the ones in whole Latin America. According to the 2015 quality of death index [18], a ranking of palliative care worldwide, palliative care in developing countries is a rare or non-existing entity. Brazil figures in the 42rd position. Nevertheless, short-term improvement is achievable. Japan raised from the 23rd position in 2010 to the 14th position in 2015 by providing better palliative care in cancer patients through a national governmental program [18].

In our survey, the importance of education on end-of-life care was consonant. Almost all of the interviewed physicians thought it should be a mandatory subject in the medical curriculum. Despite the growing interest in the issue, the importance of training, and the raising social demand, medical schools still do not acknowledge palliative care as being essential for medical education [19]. Murray et al. well defined a new approach to the conception of illness trajectory in palliative care also imbued with a perspective in patient’s wish, this shows the everchanging nature of medicine and the need to deepen the study of terminal patients, not only in end-of-life, but also throughout all the phases experienced by the Patients in palliative care [20]. A systematic review suggested that there was little consistency in medical undergraduate’s education in palliative care and that medical students were not prepared for the realities of caring for patients with a chronic progressive life-threatening illnesses [21]. In Brazil, only one-third of the medical schools have palliative care medicine as a compulsory discipline in the medical curriculum. The main reason is the lack of specialized teaching staff [22].

Our study also found out that end-of-life care is best presented during residency training. Corroborating the findings of von Gunten et al. [23]. A vast number of different teaching techniques are available but no consensus has been reached regarding the best teaching method. The use of death rounds during the residency is not only well received by trainees, providing an opportunity for reflection, but also can be easily incorporated in the medical curriculum [24]. Lester et al. showed that a one-week’s rotation of house staff is insufficient [25] while Yacht et al. reported that 1 week was sufficient for medical residents [26]. The use of clinical rotations, is cited by some, as being paramount to competency in end-of-life care [27, 28]. Even on-line educational materials addressing practical approaches in palliative care turned out to be a useful teaching tool to supplement residency curriculum [29]. A systematic review, addressing end-of-life training in US Medical Schools found out that regardless of the teaching method, an improvement was found in the medical competency in caring for the dying [30]. Similar findings were reported by a systematic review of postgraduate palliative care education. Either clinical rotations or multi-faceted interventions produced improvements in knowledge [31].

The most significant finding of our research is that Medical residency plays an important role in the overall knowledge of palliative medicine. Even without formal training in palliative medicine, the ones who have done residency performed better than the ones who haven’t. This results suggests that as the population ages and the prevalence of chronic diseases raises, medical residents have to face terminal patients on a daily basis, independent of the medical specialty. In England, 65% of general practitioners reported that they were currently providing palliative care to patients, demonstrating that the care of this kind of patient is no longer exclusive of the palliative care experts [32]. Another contribution of medical residency to the overall knowledge of palliative medicine is explained by the fact that field experience with dying patients plays a critical role in resident’s knowledge and attitudes [33–35]. Substantial changes in attitudes regarding the end-of-life practices occur during residency [36]. In a multi center survey, the majority of internal medicine residents agreed that learning from dying patients was meaningful [15]. The number of times that residents engage in palliative care situations, and the years of clinical experience have a positive influence on perceived competence. In our sample, corroborating findings by Mulder et al., caring for dying patients was a positive factor in overall knowledge [37].

Our study was conducted in the city of São Paulo, not only the most developed city in Brazil but also where the most renowned medical schools are located. However, it presented many limitations. Firsty, it was also centered in a hospital point of view instead of a home care view. Also, in Brazil, the medical curriculum can differ from state to state. Therefore, the average knowledge may have probably been overestimated. Secondly, palliative medicine has been only recently introduced in Brazil, so that older physicians know little or nothing about the subject. Most of the interviewers were newly graduated or had recently concluded a medical residency program. A possible selection bias may have overestimated the overall performance at the PCKT.

## Conclusions

In Summary, our study showed that not only that Brazilian’s physicians lack knowledge and training in end-of-life medicine, but also that training is considered to be a key factor in overall knowledge. Therefore, Brazilian medical schools and residency programs should focus on improving palliative training, especially those involving contact with terminal patients. Further studies involving end-of-life care and palliative medicine should be conducted to find more conclusive answers about the issue.

## Data Availability

The statistical analysis and questionnaire can be found as tables and figures. If any other information is needed, please contact the corresponding author to obtain the complete analysis report.

## Abbreviations

ANOVA: Analysis of variance
CAAE: Presentation Certificate for Ethical Appreciation
HIAE: Hospital Israelita Albert Einstein
PCKT: Palliative Care Knowledge Test
UNIFESP: Universidade Federal de São Paulo
